# Effect of cardamom consumption on inflammation and blood pressure in adults: A systematic review and meta‐analysis of randomized clinical trials

**DOI:** 10.1002/fsn3.3738

**Published:** 2023-10-07

**Authors:** Azadeh Heydarian, Negin Tahvilian, Hossein Shahinfar, Seyed Ali Abbas‐Hashemi, Reza Daryabeygi‐Khotbehsara, Naheed Aryaeian

**Affiliations:** ^1^ Department of Nutrition, School of Public Health Iran University of Medical Sciences Tehran Iran; ^2^ Student Research Committee, School of Public Health Iran University of Medical Sciences Tehran Iran; ^3^ Department of Nutrition, School of Public Health Shahid Sadoughi University of Medical Sciences Yazd Iran; ^4^ Nutrition and Food Security Research Center Shahid Sadoughi University of Medical Sciences Yazd Iran; ^5^ Department of Clinical Nutrition and Dietetics, Faculty of Nutrition Sciences and Food Technology National Nutrition and Food Technology Research Institute, Shahid Beheshti University of Medical Sciences Tehran Iran; ^6^ Institute for Physical Activity and Nutrition (IPAN) Deakin University Geelong Vitoria Australia

**Keywords:** blood pressure, cardamom, clinical trials, inflammation, meta‐analysis

## Abstract

Cardamom has the potential to offer anti‐inflammatory and antihypertensive advantages, but the findings from clinical trials have been inconsistent. To address this knowledge gap, the present systematic review and meta‐analysis were conducted to evaluate the anti‐inflammatory and antihypertensive effects of cardamom in adults. We systematically searched databases including PubMed, Scopus, and ISI Web of Sciences, for papers published up to October 2022 to identify clinical studies. Eight eligible studies were included in the meta‐analysis. A fixed model was used to estimate weighted mean difference (WMD), standardized mean difference (SMD), and 95% confidence interval (95% CI). The results showed that cardamom significantly reduced the levels of inflammatory factors, including hs‐CRP (SMD: −0.60 mg/dL; 95% CI: −0.78 to 0.42), IL‐6 (WMD: −1.25 mg/dL; 95% CI: −1.48 to −1.03), TNF‐α (WMD: −2.10 kg; 95% CI: −2.36 to −1.84, *p* < .001), and measures of systolic (WMD: −0.54 mmHg, 95% CI: −0.88, −0.19, *p* = .002) and diastolic (WMD: −0.90 mmHg; 95% CI: −1.07 to −0.73) blood pressure. The current meta‐analysis showed that cardamom can help reduce inflammation and improve blood pressure. However, due to the limited number of studies, caution must be exercised when interpreting the current results.

## Graphical abstract

Cardamom reduces the levels of inflammatory factors such as hs‐CRP, IL‐6, and TNF‐a, as well as systolic and diastolic blood pressure.

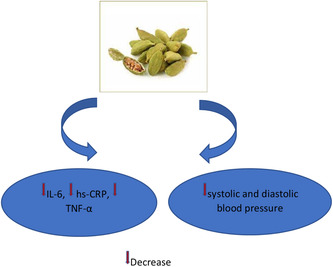

## 1 INTRODUCTION

Inflammation is the immune system's protective response against damage to the body, which causes the release of inflammatory factors such as tumor necrosis factor‐alpha (TNF‐α), interleukin‐6 (IL‐6), and high‐sensitivity C‐reactive protein (hs‐CRP). However, chronic inflammation leads to conditions such as diabetes (Zatterale et al., 2020), rheumatoid arthritis (Moosavian et al., 2020), neurodegenerative diseases (Zhang et al., 2020), cancer (Murata, 2018), and cardiovascular diseases (Asbaghi et al., 2021; Soysal et al., 2020). Studies have shown that inflammation, along with an unhealthy diet and lifestyle, as well as disorders in the peripheral vascular and sympathetic nervous system, may contribute to high blood pressure (Zhao et al., 2023). Elettaria cardamomum, also known as green cardamom, is a member of the Zingiberaceae family. This plant is used as an aromatic spice (Singletary, 2022). Cardamom grows mostly in Indonesia, India, Pakistan, Burma, Bangladesh, and Asia. Cardamom offers health benefits through its anti‐oxidantive, antimutagenic (Saeed et al., 2014), antibacterial, anti‐inflammatory (Souissi et al., 2020), antidiabetic (Razzaque et al., 2021), cardioprotective (Goyal et al., 2015), and hepatoprotective properties (Elguindy et al., 2016; Yahyazadeh et al., 2021). The biologically active compounds of cardamom include 1,8‐cineole, α‐terpinyl acetate (the maximum amount), sabinene, linalool acetate, nerolidol, thujene, pinene, cymene, limonene, geranial, and myrcene (Ahmad et al., 2022). Cardamom has been shown to reduce inflammation by preventing lipid peroxidation and acting as a scavenger of free radicals. Furthermore, cardamom exerts its anti‐inflammatory effects by blocking inflammatory pathways such as cyclooxygenase‐2 (COX‐2), inducible nitric oxide synthase (i‐NOS), and nuclear factor‐κB (NF‐κB) (Kandikattu et al., 2017). 1,8‐cineol, the dominant compound in cardamom, has been shown to have antibacterial, antioxidant, and anti‐inflammatory effects. It also prevents the release of inflammatory factors and reactive oxygen species (ROS). The reduction in blood pressure by 1,8‐cineol might be achieved through the regulation of nitric oxide (NO) production and the improvement of cardiac systolic function (Cai et al., 2021; Farhanghi et al., 2022). Moreover, it has also been reported in animal models that cardamom reduces blood pressure through vasorelaxation effects (Kanthlal et al., 2020). Verma et al. (2009) showed that a daily intake of 3 g cardamom in subjects with stage 1 hypertension led to a significant reduction in blood pressure after 12 weeks of intervention. A clinical trial among patients with nonalcoholic fatty liver disease showed that consuming 3 g of cardamom over a period of 3 months reduced the levels of inflammatory factors, including TNF‐α, IL‐6, and hs‐CRP (Daneshi‐Maskooni et al., 2018). In contrast, a trial involving 204 patients with type 2 diabetes mellitus found that cardamom did not have a significant impact on sICAM‐1, as well as systolic and diastolic blood pressures (SBP and DBP) (Azimi et al., 2016). Another study conducted in patients with type 2 diabetes showed that consuming cardamom for 8 weeks did not result in significant changes in hs‐CRP levels (Azimi et al., 2014).

Due to conflicting findings, the impact of cardamom on inflammation and blood pressure remains uncertain. Therefore, we conducted a comprehensive systematic review and meta‐analysis of clinical trials on adults to summarize the potential antihypertensive and anti‐inflammatory effects of cardamom.

## 2 METHODS

The systematic review and meta‐analysis protocol have been registered with the International Prospective Register of Systematic Reviews (PROSPERO), with the assigned number CRD42022365939. A systematic literature search was conducted following the guidelines of the Reporting Items for Systematic Reviews and Meta‐analysis (PRISMA‐2020) (Page et al., 2021).

### 2.1 Search strategy

Two authors (AH & NT) independently conducted systematic searches of databases, including PubMed, Scopus, and ISI Web of Science, up to October 2022, to identify and select studies. Related search terms were used to identify studies published in the English language (Data S1). Furthermore, we conducted a comprehensive manual search and cross‐checked the references of the included studies to ensure that we did not overlook any relevant articles.

### 2.2 Eligibility criteria

Inclusion criteria were as follows: (a) randomized clinical trials (RCTs) with a parallel or crossover design, (b) involving individuals aged 18 or older, and (c) studies that reported mean ± SD for inflammatory biomarkers, systolic blood pressures (SBPs), and diastolic blood pressures (DBPs) before and after the administration of cardamom in intervention and control group. We excluded letters, comments, reviews (including systematic and meta‐analyses), experimental animal studies, studies involving pregnant women and children, unpublished dissertations, gray literature, patents, congress abstracts, and studies without a control group.

### 2.3 Data extraction

After the study selection process, two independent reviewers conducted data extraction. The following information was systematically extracted from each included randomized trial: study design, the surname of the first author, study location, publication year, sample size, participants' gender, mean age, intervention details (including dose, type, and duration), health status of participants, changes in mean and SD of BMI, inflammatory biomarkers, and SBPs and DBPs from pre‐ to postintervention (Table 1).

**TABLE 1:** Characteristics of the included studies.

Studies	Country	Study design	Participant	Sex	Sample size			Trial duration (week)	Means age		Means BMI		Intervention		
						IG	CG		IG	CG	IG	CG	Type intervention	Dose (mg/day)	Control group
Daneshi‐Maskooni et al. (2018)	Iran	Parallel, R, PC, DB	NAFLD‐ overweight or obese	M/F	87	43	44	12	45.5 ± 8.9	45 ± 7.7	30.5 ± 2.4	30.7 ± 3.2	Green cardamom	3000	Placebo
Cheshmeh et al. (2022)	Iran	Parallel, R, PC, DB	Polycystic ovary syndrome–obese	F	197	99	95	16	32.99 ± 5.57	33.81 ± 5.42	34.78 ± 3.39	35.18 ± 5.16	Green cardamom low‐calorie diet	3000	Low‐calorie diet‐placebo
Kazemi et al. (2017)	Iran	Parallel, R, PC, DB	hyperlipidemic, overweight, obese, and prediabetic	F	80	40	40	8	48.3 ± 10.4	47.5 ± 10.3	29.7 ± 4.04	29.3 ± 3.1	Green cardamom powder	3000	Placebo
Ghazi Zahedi et al. (2021)	Iran	Parallel, R, PC, DB	T2DM‐ overweight or obese	M/F	83	41	42	10	NR	NR	NR	NR	Dried fruits of Elettaria cardamom	3000	Placebo
Azimi et al. (2014)	Iran	Parallel, R, CO, SB	T2DM	M/F	81	42	39	8	51.59 ± 8.42	53.64 ± 8.11	28.96 ± 1.29	28.4 ± 1.24	Green cardamom– black tea	3000	Black tea
Zahedi et al. (2022)	Iran	Parallel, R, PC, DB	T2DM	M/F	83	41	42	10	54 ± 5.55	53 ± 4.44	29 ± 3.85	29 ± 3.11	Green cardamom	3000	Placebo
Fatemeh et al. (2017)	Iran	Parallel, R, PC, DB	Prediabetic, overweight, and obese	F	80	40	40	8	48.3 ± 10.4	47.5 ± 10.3	29.7 ± 4.04	29.3 ± 3.1	Green cardamom powder	3000	Placebo
Azimi et al. (2016)	Iran	Parallel, R, CO, SB	T2DM	M/F	81	42	39	8	51.59 ± 8.42	53.64 ± 8.11	28.96 ± 1.29	28.40 ± 1.24	Green cardamom–black tea	3000	Black tea

Abbreviations: BMI, body mass index; CG, control group; CO, control; DB, double blind; F, female; IG, intervention group; M, male; PC, placebo controlled; R, randomized; SB, single blind.

### 2.4 Quality assessment of studies

The Revised Cochrane Risk‐Of‐Bias tool for randomized trials (RoB 2) was used to evaluate the quality of studies. Several methodological aspects were considered for the assessment. These include random sequence generation, allocation concealment, blinding of participants and personnel, blinding of outcome assessments, incomplete outcome data, selective reporting, and other potential threats to validity. Studies were stratified into low, high, or unclear risk of bias for each domain (Table 2). Disagreements were resolved through arbitration or consensus.

**TABLE 2:** Risk of bias assessment.

Studies	Random sequence generation	Allocation concealment	Selective reporting	Other sources of bias	Blinding (participants and personnel)	Blinding (outcome assessment)	Incomplete outcome data	General risk of bias
Daneshi‐Maskooni et al. (2018)	L	L	L	L	L	U	L	Low
Cheshmeh et al. (2022)	L	L	L	L	L	U	L	Low
Kazemi et al. (2017)	L	L	L	L	L	U	L	Low
Ghazi Zahedi et al. (2021)	L	L	L	L	L	U	L	Low
Azimi et al. (2014)	L	U	L	L	H	U	L	Low
Zahedi et al. (2022)	L	L	L	U	L	U	L	Low
Fatemeh et al. (2017)	L	L	L	L	L	U	L	Low
Azimi et al. (2016)	L	U	L	L	H	U	L	Low

*Note*: General Low Risk < 2 high risk, General moderate risk = 2 high risk, General high risk > 2 high risk.

Abbreviations: H, High; L, Low; M, Moderate; U, Unclear.

### 2.5 Quantitative data synthesis and statistical analysis

Mean and SD of the baseline and postintervention measures were used in both the intervention and control groups to assess the effects of cardamom on inflammatory markers, including TNF‐α, IL‐6, and hs‐CRP, as well as on blood pressure measures (i.e., SBPs and DBPs).

In the studies that did not report mean and SD, available statistics were converted into mean and SD using a proper formula: SD difference = square root [(SD pretreatment)2 + (SD posttreatment)2 − (2 × *R* × SD pretreatment × SD posttreatment)]. A conservative correlation coefficient (*R*) estimate (0.9) was used (Borenstein et al., 2021). The following formula was employed to convert standard error of the mean (SEM) to SD: SD = SEM × √*n*, being “*n*” the number of subjects in each group. Medians and interquartile ranges were converted to mean and SD using the following formula: SD = IQR/1.35 (symmetrical data distribution) (Hozo et al., 2005). To ascertain the relationship with forest plots, we used a fixed‐effects model. The I‐squared (*I* ^2^) statistic was used to estimate heterogeneity. Evidence of heterogeneity was confirmed when *I* ^2^ was greater than 50% with a *p*‐value <.1 (Cochran, 1954). To discern the potential sources of heterogeneity, we performed a subgroup analysis based on the duration of intervention and sex. To estimate the pooled effect size, a sensitivity analysis was performed. Eggers' regression symmetry test and visual funnel plots were used to investigate potential publication biases (Egger et al., 1997; Peters et al., 2008). Quantitative analysis of homogeneity and heterogeneity was performed using the *I* ^2^ statistic and *χ* ^2^ test (Higgins et al., 2019). STATA software version 16.1 was used to perform the statistical analyses. A *p*‐value of <.05 was considered to be statistically significant.

## 3 RESULTS

### 3.1 Study selection

**FIGURE 1:**
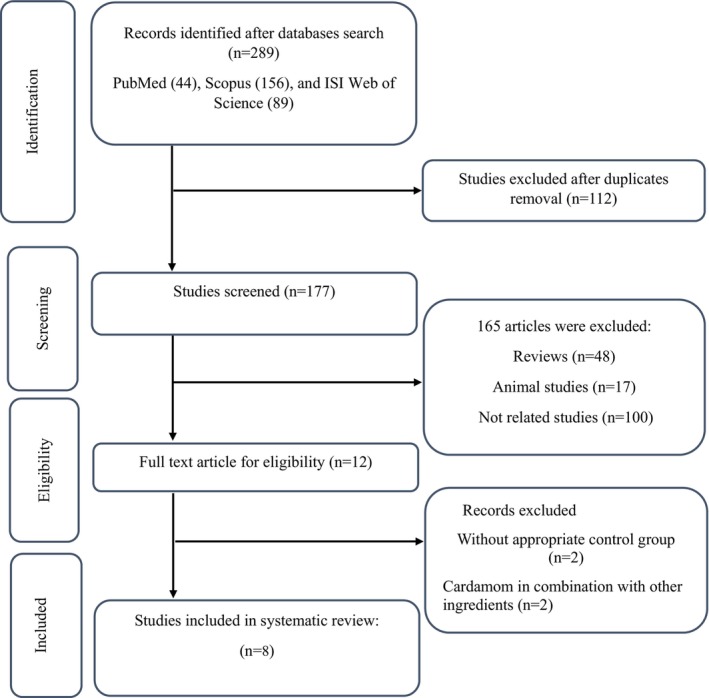
Flow chart of study selection for inclusion trials in the systematic review.

The primary database search strategy identified a total of 289 studies (44 records from PubMed, 156 from Scopus, and 89 from Web of Science). After removing 112 duplicated records, 177 articles underwent title and abstract screening to identify eligible studies, and 165 articles were excluded. The full text of the remaining 12 studies was assessed, and 8 articles were eventually included in the current study (Azimi et al., 2014, 2016; Cheshmeh et al., 2022; Daneshi‐Maskooni et al., 2018; Fatemeh et al., 2017; Ghazi Zahedi et al., 2021; Kazemi et al., 2017; Zahedi et al., 2022) (Figure 1).

### 3.2 Study characteristics

General characteristics of the included studies are presented in Table 1. All of the included studies were RCTs conducted in Iran and involved a total of 769 participants. The intervention duration ranged from 8 to 16 weeks, and dosage of the supplemented green cardamom in the included studies was 3000 mg (Azimi et al., 2014, 2016; Cheshmeh et al., 2022; Daneshi‐Maskooni et al., 2018; Fatemeh et al., 2017; Ghazi Zahedi et al., 2021; Kazemi et al., 2017; Zahedi et al., 2022). Five studies included both sexes, while three studies were conducted exclusively among females (Cheshmeh et al., 2022; Fatemeh et al., 2017; Kazemi et al., 2017). Four studies included individuals with type 2 diabetes (Azimi et al., 2014, 2016; Ghazi Zahedi et al., 2021; Zahedi et al., 2022), one study included overweight and obese individuals with prediabetes (Fatemeh et al., 2017), one study included overweight and obese people who were hyperlipidemic and had prediabetes (Kazemi et al., 2017), one included obese individuals with polycystic ovary syndrome (Cheshmeh et al., 2022), and one study included overweight or obese individuals with nonalcoholic fatty liver disease (Daneshi‐Maskooni et al., 2018). [corrections added on 26 October 2023, after the first online publication: Yaghooblou et al. (2015) was replaced by Fatemeh et al. (2017).]

### 3.3 Meta‐analysis

#### 3.3.1 Effect of cardamom on SBP

**FIGURE 2:**
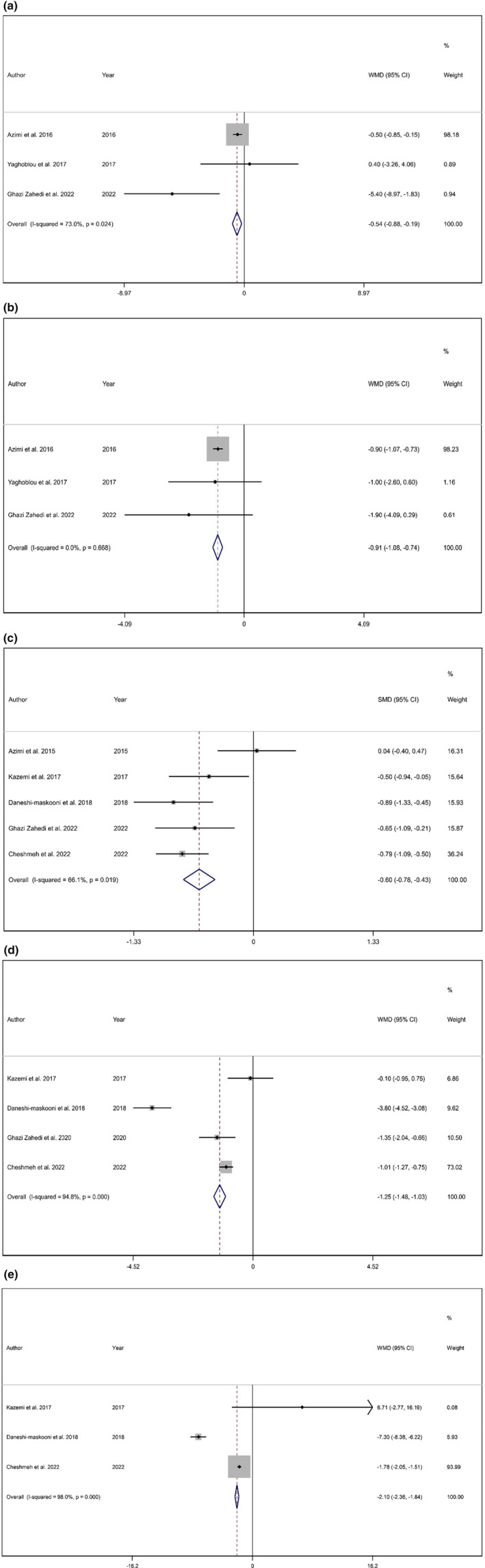
Forest plot detailing weighted mean difference and 95% confidence intervals (CIs) for the effect of cardamom consumption on (a) SBP (mmHg); (b) DBP (mmHg); (c) hs‐CRP; (d) IL‐6; and (e) TNF‐α.

The overall effect showed a significant reduction in SBP (WMD: −0.54 mmHg, 95% CI: −0.88, −0.19, *p* = .002) with a significance level of heterogeneity (*I* ^2^ = 73.0%; *p* = .024) (Figure 2a).

#### 3.3.2 Effect of cardamom on DBP

The overall pooled effect size showed a significant reduction in DBP (WMD: −0.90 mmHg; 95% CI: −1.07 to −0.73, *p* < .001) with no significant heterogeneity (*I* ^2^ = 0.0%, *p* = .668) (Figure 2b).

#### 3.3.3 Effect of cardamom on hs‐CRP

Combining the results of five studies indicated that cardamom intake statistically reduces hs‐CRP levels (SMD: −0.60 mg/dL; 95% CI: −0.78 to 0.42, *p* < .001) with significant heterogeneity (*I* ^2^ = 66.1%, *p* = .019) (Figure 2c). However, subgroup analysis showed that studies with a duration longer than or equal to 10 weeks resulted in significant changes (WMD: −0.78, 95% CI: −0.99, −0.57, *p* < .001). Moreover, studies conducted predominantly among females might be the source of heterogeneity (Table 3).

**TABLE 3:** Subgroup analyses of cardamom consumption on inflammatory factors in adults.

	Number of studies	WMD (95% CI)	*p*‐Value	*p*‐Value heterogeneity	*I* ^2^ (%)	*p* for between‐subgroup heterogeneity
Subgroup analyses of cardamom consumption on IL6						
Trial duration (week)						
≤10	2	−0.856 (−1.393, −0.319)	.002	.026	79.9	.112
>10	2	−1.335 (−1.581, −1.088)	<.001	<.001	98.0	
Sex						
Female	2	−0.932 (−1.182, −0.681)	<.001	.046	74.9	<.001
Female/male	2	−2.521 (−3.020, −2.022)	<.001	<.001	95.7	
Subgroup analyses of cardamom consumption on hs‐CRP						
Trial duration (week)						
<10	2	−0.224^a^ (−0.535, 0.088)	.159	.094	64.3	.004
≥10	3	−0.783^a^ (−0.997, −0.570)	<.001	.753	0.0	
Sex						
Female	2	−0.703^a^ (−0.948, −0.459)	<.001	.273	16.7	.253
Female/male	3	−0.498^a^ (−0.752, −0.244)	<.001	.010	78.5	

^a^ Standardized mean difference.

#### 3.3.4 Effect of cardamom on IL‐6

According to the results of the meta‐analysis, the consumption of cardamom has been found to significantly decrease IL‐6 levels (WMD: −1.25 mg/dL; 95% CI: −1.48 to −1.03, *p* < .001) with significant heterogeneity (*I* ^2^ = 94.8%, *p* < .001) (Figure 2d). Subgroup analysis revealed gender and duration as sources of heterogeneity (Table 3).

#### 3.3.5 Effect of cardamom on TNF‐α

The pooled effect size from three studies showed a significant reduction in TNF‐α (WMD: −2.10 kg; 95% CI: −2.36 to −1.84, *p* < .001). Moreover, a significant heterogeneity (*I* ^2^ = 98.0%, *p* < 0.001) was observed. The effect of cardamom on TNF‐α is presented in Figure 2e.

#### 3.3.6 Publication bias and sensitivity analysis

**FIGURE 3:**
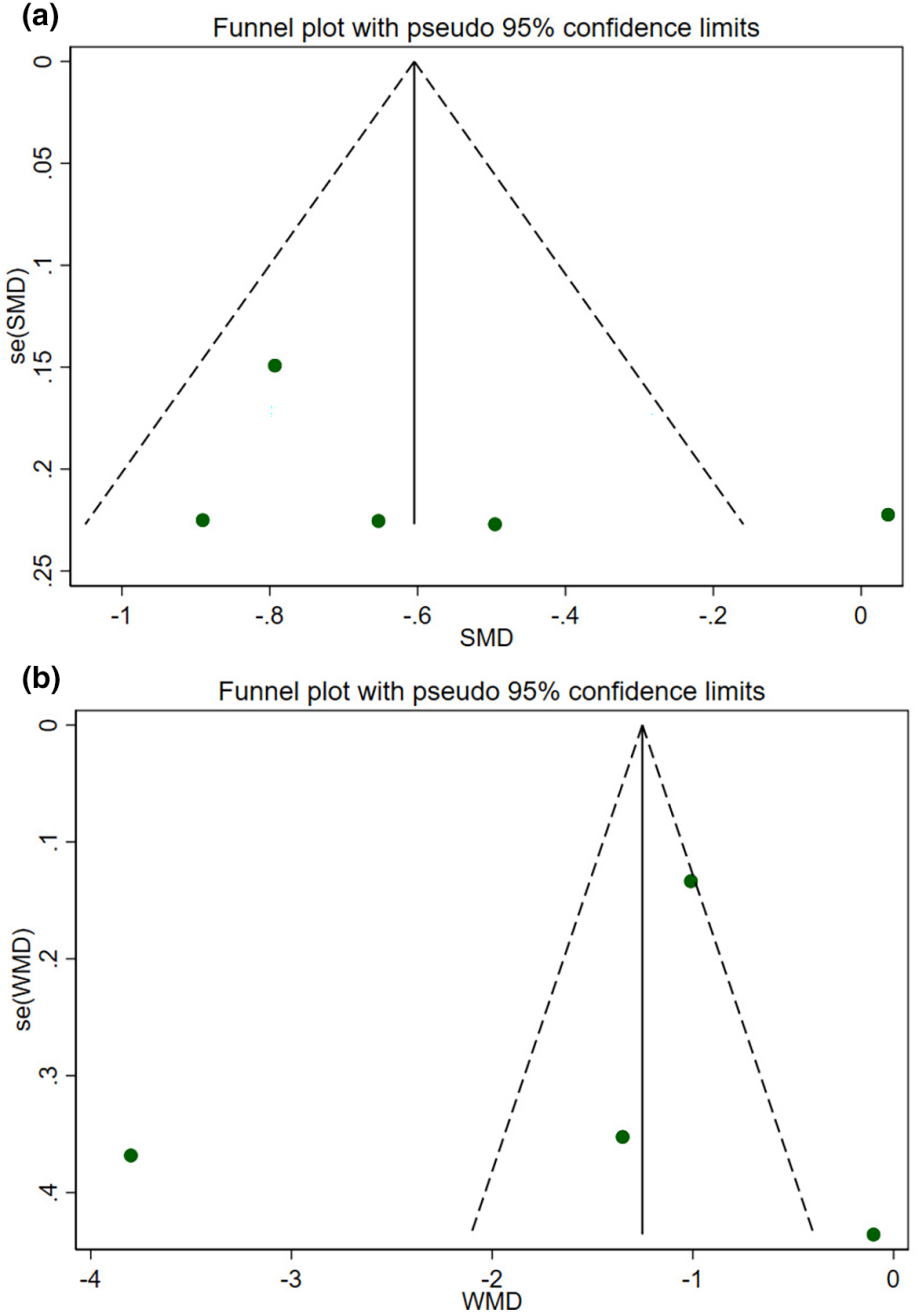
Funnel plots for the effect of cardamom consumption on (a) hs‐CRP; (b) IL‐6.

To determine the impact of each trial on the pooled effect size, we conducted a sensitivity analysis. The sensitivity analysis for hs‐CRP and IL‐6 showed that the overall estimates were not affected by the elimination of any of the included studies. We also examined publication bias by visually inspecting the funnel plot and conducting Egger's weighted regression tests. The results of the funnel plot (Figure 3) and Egger's test showed no publication bias for hs‐CRP (*p* = .459) and IL‐6 (*p* = .627).

## 4 DISCUSSION

For centuries, cardamom has been used in traditional medicine to treat a variety of conditions such as tooth and gum infections, asthma, kidney and digestive disorders, diarrhea, nausea, cataracts, and cardiac disorders (Ashokkumar et al., 2020). According to a recent narrative review, *Elettaria Cardamomum* (green cardamom) may have lipid‐modifying, antihypertensive, anti‐inflammatory, antioxidant, and antithrombotic properties and these are thought to be attributed to the presence of polyphenol compounds (Yahyazadeh et al., 2021). In this systematic review and meta‐analysis, we summarized the results of clinical trials assessing the effect of *Elettaria Cardamomum* (green cardamom) on inflammatory indices and blood pressure measures. The current study showed that supplementation with green cardamom reduces the levels of inflammatory factors, including TNF‐α, hs‐CRP, and IL‐6, as well as blood pressure measures, including SBP and DBP. Subgroup analyses showed a significant reduction in IL‐6 for both study duration (≤10 vs. >10 weeks) and sex (male vs. female). Additionally, subgroup analyses showed that although hs‐CRP did not change significantly in studies that lasted less than 10 weeks, a significant reduction was observed in studies conducted for greater or equal to 10 weeks. Moreover, subgroup analysis based on sex (male vs. female) showed a significant reduction in the level of hs‐CRP. In line with the current results, a clinical trial reported that 3 g of cardamom combined with a low‐calorie diet over 16 weeks reduced serum levels of inflammatory factors including TNF‐α, IL‐6, and hs‐CRP (Cheshmeh et al., 2022). In another RCT, Winarsi and Susilowati (2018) found that consuming a drink rich in antioxidant rhizome cardamom (Fd‐Carrhi) for 2 months reduced IL‐6 and CRP levels in women with atherosclerosis. Green cardamom potentially reduces inflammation by downregulating the expression of COX‐2 and iNOS and decreasing inflammatory cytokines such as IL‐6 and TNF‐α. Furthermore, cardamom improves oxidative stress through the regeneration of antioxidant enzymes such as superoxide dismutase (SOD), catalase (CAT), and reduced glutathione (GSH) (Kandikattu et al., 2017; Sengupta et al., 2005). One of the major compounds in cardamom is 1,8‐Cineole, which possesses anti‐inflammatory and antioxidant properties, as well as protective effects against cardiovascular disease. Also, 1,8‐cineole inhibits NF‐κB phosphorylation and its transfer into the nucleus, regulating the expression of important transcription factors such as NF‐κB and Nrf2, which can potentially regulate the inflammatory response (Cai et al., 2021). In line with the current findings on blood pressure measures, a single‐arm study conducted by Mohammed and Mohammed (2020) showed that daily consumption of 3 g of cardamom for 3 months led to a decrease in SBP and DBP among patients with stage 1 hypertension. Another single‐arm study investigated the impact of cardamom on blood pressure in patients with stage 1 hypertension and found that supplementing with 3 g of cardamom powder for 3 months significantly reduced SBP and DBP as well as the mean blood pressure (Verma et al., 2009). In contrast, Azimi et al. (2014) reported that consumption of cardamom, cinnamon, ginger, and saffron along with tea for 8 weeks did not result in significant changes in blood pressure measures in people with type 2 diabetes. Various mechanisms have been suggested to explain how cardamom can help regulate blood pressure. These mechanisms include acting as a calcium channel antagonist, promoting vasodilation through cholinergic activity, stimulating the release of nitric oxide from the endothelium, and having a diuretic effect (Gilani et al., 2008). Moreover, based on previous studies, angiotensin II contributes to the development of cardiovascular diseases and blood pressure regulation. Another potential mechanism is the role of 1,8‐cineole in suppressing angiotensin. In animal models of hypertension, cineole has been shown to decrease SBP and increase plasma nitrite levels (Moon et al., 2014). In this meta‐analysis, although we conducted a comprehensive search and covered most of the main databases, there is still a possible risk of publication bias. There are some limitations to consider. All included studies were conducted in Iran. Therefore, the current results may not be generalizable to other communities. Furthermore, the results of this study should be interpreted with caution due to significant heterogeneity among the included studies. However, subgroup analyses were used to reduce heterogeneity.

## 5 CONCLUSION

In conclusion, the current systematic review and meta‐analysis provide convincing evidence in favor of the effectiveness of green cardamom supplementation in improving inflammatory markers and blood pressure.

## AUTHOR CONTRIBUTIONS

**Azadeh Heydarian:** Data curation (equal); investigation (equal); methodology (equal); writing – original draft (equal); writing – review and editing (equal). **Negin Tahvilian:** Data curation (equal); investigation (equal); methodology (equal); writing – original draft (equal); writing – review and editing (equal). **Hossein Shahinfar:** Formal analysis (equal); software (equal). **Seyed Ali Abbas‐Hashemi:** Data curation (equal); writing – review and editing (equal). **Reza Daryabeygi‐Khotbehsara:** Writing – review and editing (equal). **Naheed Aryaeian:** Project administration (equal); supervision (equal); writing – review and editing (equal).

## CONFLICT OF INTEREST STATEMENT

The authors declare no conflicts of interest.

## Supporting information


Data S1.

## Data Availability

On reasonable request, the corresponding author will provide the datasets created and used in the current study.
