# Supplementary material for: Effect of cardamom consumption on inflammation and blood pressure in adults: A systematic review and meta‐analysis of randomized clinical trials
Source: Food Sci Nutr. 2023 Oct 7;12(1):3–12. doi: 10.1002/fsn3.3738 (PMC10804083; doi:10.1002/fsn3.3738)
Supplement: Supplementary file 1 — Data S1. [file FSN3-12-3-s001.docx]

"cardamom" OR "Elettaria cardamomum" OR " cardamomum" OR Elettarias OR Cardamoms OR Cardamon OR Cardamons OR "Amomum cardamomum" OR "Amomum cardamomums" OR "cardamomums, Amomum" OR "cardamom, Elettaria" OR "Elettaria cardamomums" AND intervention OR "controlled trial" OR randomised OR randomized OR random OR randomly OR placebo OR "clinical trial" OR trial OR "randomized controlled trial" OR "randomized clinical trial" OR "randomized, placebo-controlled trials" OR RCT OR blinded OR "double blind" OR "double blinded" OR trials OR "Cross-Over Studies" OR "Cross-Over" OR "Cross-Over Study" OR parallel.
